# Dataset describing the growth pattern, amino acid and fatty acid profile of five indigenous marine microalgae species of Bangladesh

**DOI:** 10.1016/j.dib.2022.108643

**Published:** 2022-09-28

**Authors:** Jakia Hasan, Zahidul Islam, Ahmad Fazley Rabby, Saima Sultana Sonia, Md. Aktaruzzaman, Turabur Rahman, Shafiqur Rahman, Yahia Mahmud

**Affiliations:** a Marine Fisheries and Technology Station, Bangladesh Fisheries Research Institute, Cox's Bazar 4700, Bangladesh; b Bangladesh Fisheries Research Institute, Mymensingh 2201, Bangladesh

**Keywords:** Indigenous microalgae, Growth pattern, Essential amino acid, Fatty acid, Polyunsaturated fatty acid, Bangladesh

## Abstract

This paper presents the data on the growth pattern, amino acid, and fatty acid profile of five (5) selected indigenous marine microalgae (*Chaetoceros* sp.; *Isochrysis* sp.; *Skeletonema* sp.; *Nannochloropsis* sp.; and *Tetraselmis* sp.) of Bay of Bengal, Bangladesh. The microalgae species were cultured in f/2 Guillard's medium with maintaining standard physico-chemical parameters. The growth pattern was determined for all the microalgae as a prerequisite for further necessary experimental works. All the species were mass cultured using the same culture medium and harvested (centrifuging method), and dried (60 ℃ for 12 h) at their stationary phase. Finally, the amino acid and fatty acid analyses were performed. In many contexts, the amino acid and fatty acid data showed significant differences (*p* < 0.05) among these experimental species. However, by understanding these experimental species' nutritional profiles, one can easily choose the desired one that is most appropriate for their intended application.

**Specifications Table**

**Table:** 

Subject	Agricultural Sciences, Aquatic Science
More specific subject area	Microalgae, Algal-biodiversity, Nutrition science
Type of data	Table, chart, and figure
How data were acquired	Growth pattern was observed through cell count and biomass measurement. Morphological traits were identified using a computer based light microscope. Amino acid analysis was done through SYKAM amino acid analyzer. Fatty acid analysis was done through gas chromatographic mass spectrophotometry (GCMS).
Data format	Raw and analyzed primary data
Description of data collection	For morphological trait: Microscopic observation For growth pattern: cell count and biomass. For water quality: temperature, pH, salinity, dissolve oxygen, total ammonia nitrogen, nitrite nitrogen, and soluble reactive phosphorus. For amino acid: SYKAM amino acid analysis of aliquot HCl extract from microalgae. For fatty acid: GCMS analysis of extracted oil from microalgae.
Data source location	Live Feed Laboratory, Marine Fisheries and Technology Station, Bangladesh Fisheries Research Institute, Cox's Bazar-4700, Bangladesh.
Data accessibility	Data are available with this article and also at https://doi.org/10.17632/6YHWWT57YP.2

## Value of the Data

• Providing the presence and its percentages in the amount of amino acid and fatty acid content in the selected five indigenous microalgae species with mentioning essential and non-essential classifications.
• Providing a basis in complete understanding of the nutritional profiles (amino acid, fatty acid) of these selected commercially important microalgae species of Bay of Bengal, Bangladesh.
• Understanding the differences in growth pattern, amino acid, and fatty acid among the commercially important different microalgae species which will help in choosing the best-suited microalgae species for a definite use.
• Fatty acid data and amino acid data will be useful to select suitable microalgae species as biofuel production raw material as well as to select suitable microalgae species as animal feedstuff.

## 1 Data Description

**Fig. 1:**
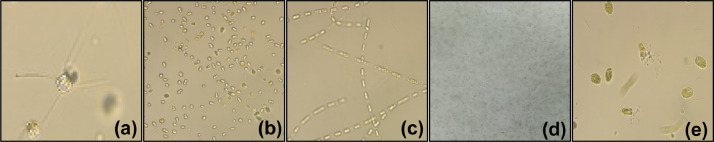
Experimental microalgae species. (a) *Chaetoceros* sp. (b) *Isochrysis* sp. (c) *Skeletonema* sp. (d) *Nannochloropsis* sp. (e) *Tetraselmis* sp.

The raw data on growth pattern, amino acid and fatty acid profile of five indigenous marine microalgae species of Bangladesh are as in [1]. Microalgae have recently attracted considerable interest worldwide due to their potential applications in renewable energy, biopharmaceuticals, and nutraceuticals. Fig. 1 shows the experimental indigenous marine microalgae species collected from previously preserved samples at Marine Fisheries and Technology Station (MFTS), Bangladesh Fisheries Research Institute (BFRI), Cox's Bazar, Bangladesh.

Among the five (5) selected species, *Chaetoceros* sp., *Isochrysis* sp., and *Skeletonema* sp., were brown microalgae whereas *Nannochloropsis* sp., and *Tetraselmis* sp. were green microalgae. All of the species were identically distinct from one another in terms of their physical characteristics (Table. 1).

**Table 1:** Morphological traits of the selected microalgae species.

	Species				
Traits	*Chaetoceros* sp.	*Isochrysis* sp.	*Skeletonema* sp.	*Nannochloropsis* sp.	*Tetraselmis* sp.
Color	Brown	Brown	Brown	Green	Green
Shape	Elliptic	Round	Cylindrical	Round-spherical	Oval
Length	4.2 ± 0.3 µm	4.5 ± 0.2 µm	3.1 ± 0.2 µm	6.2 ± 0.4 µm	9.9 ± 0.4 µm
Flagella	Yes	Yes	Yes	No	Yes
Motility	Yes	No	No	No	Yes
Chain formation	Yes	No	Yes	No	No

Values are mean ± standard error of triplicate measurements.

**Fig. 2:**
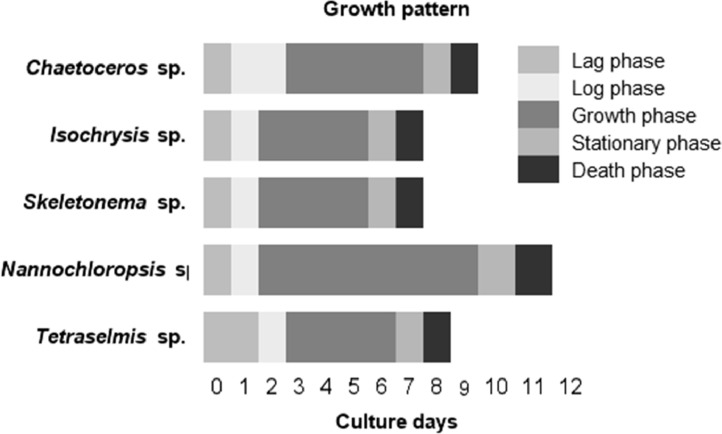
Growth pattern of the selected microalgae species.

Growth phases of the selected microalgae in F/2 Guillards's culture medium are shown in this data (Fig. 2). The stationary and death phases of the selected species started at different distinct times (6–11 days).

This data shows *Skeletonema* sp. had significantly (*p* < 0.05) higher growth rates and biomass production ability, whereas *Nannochloropsis* sp. had significantly (*p* < 0.05) higher cell density compared to all other species (Table 2).

**Table 2:** Growth dynamics of the selected microalgae species.

	Characteristics		
Species	Growth rates (µ/day)	Maximum cell density (× 10^6^ cells/mL)	Biomass (g/L/day)
*Chaetoceros* sp.	0.41 ± 0.07^a^	4.92 ± 0.15^c^	0.024 ± 0.00^b^
*Isochrysis* sp.	0.34 ± 0.04^b^	4.55 ± 0.22^c^	0.029 ± 0.00^b^
*Skeletonema* sp.	0.49 ± 0.03^a^	5.13 ± 0.19^b^	0.036 ± 0.00^a^
*Nannochloropsis* sp.	0.22 ± 0.03^c^	7.66 ± 0.36^a^	0.019 ± 0.00^c^
*Tetraselmis* sp.	0.53 ± 0.09^a^	4.91 ± 0.61^bc^	0.025 ± 0.00^b^

Values are mean ± standard error of triplicate measurements; Different letters used in each column demonstrate the significant difference (*p* < 0.05).

Physico-chemical parameters such as temperature, light intensity, salinity, pH, and total ammonium nitrogen, nitrite-nitrogen, and soluble reactive phosphorus data of the culture medium during the experimental period are shown in Table 3. In this case, dissolve oxygen, pH, total ammonium nitrogen, and soluble reactive phosphorus showed some significant (*p* < 0.05) differences among some cultures, whereas the rest of the parameters didn't differ significantly (*p* > 0.05) during the experimental period.

**Table 3:** Physico-chemical parameters of the cultured medium during culture period (up to stationary phase).

		Species				
Parameters	Unit	*Chaetoceros* sp.	*Isochrysis* sp.	*Skeletonema* sp.	*Nannochloropsis* sp.	*Tetraselmis* sp.
Temperature	⁰C	26.60 ± 0.49^a^	26.73 ± 0.52^a^	26.73 ± 0.52^a^	26.80 ± 0.55^a^	26.60 ± 0.49^a^
Light intensity	µEm^−2^s^−1^	150.0 ± 0.00^a^	150.0 ± 0.00^a^	150.0 ± 0.00^a^	150.0 ± 0.00^a^	150.0 ± 0.00^a^
Salinity	Ppt	29.33 ± 0.33^a^	28.33 ± 0.33^a^	30.67 ± 0.33^a^	30.00 ± 0.58^a^	29.67 ± 0.67^a^
Dissolve oxygen	mg/L	6.70 ± 0.12^a^	6.53 ± 0.03^bc^	6.60 ± 0.06^ab^	6.43 ± 0.08^c^	6.43 ± 0.07^c^
pH	-	8.25 ± 0.06^b^	8.54 ± 0.07^a^	8.34 ± 0.07^b^	8.29 ± 0.09^b^	8.30 ± 0.09^b^
Total ammonia nitrogen	mg/L	0.71 ± 0.01^ab^	0.67 ± 0.01^b^	0.63 ± 0.00^b^	0.61 ± 0.00^b^	0.57 ± 0.00^b^
Nitrite nitrogen	mg/L	0.65 ± 0.01^a^	0.62 ± 0.01^a^	0.62 ± 0.00^a^	0.63 ± 0.00^a^	0.60 ± 0.00^a^
Soluble reactive phosphorus	mg/L	0.17 ± 0.00^a^	0.13 ± 0.00^b^	0.15 ± 0.00^b^	0.15 ± 0.00^b^	0.13 ± 0.00^b^

Values are mean ± standard error of triplicate measurements; Different letters used in each column demonstrate the significant difference (*p* < 0.05).

**Fig. 3:**
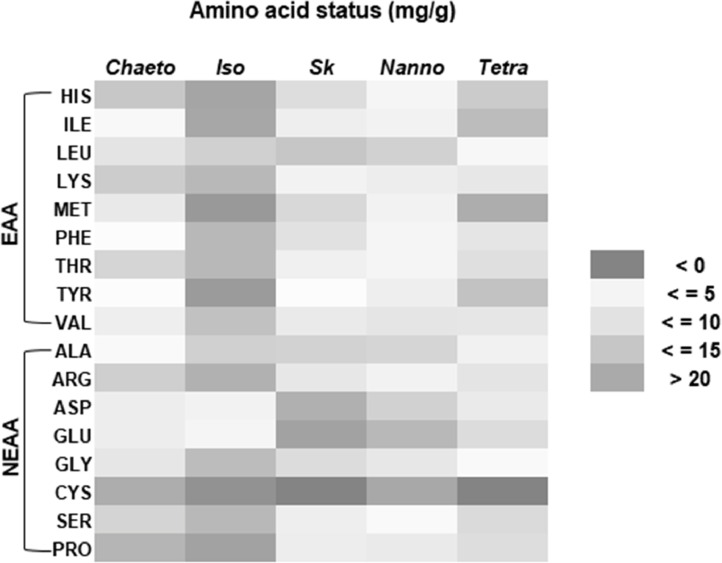
Heat-map presentation of amino acid content in the cultured microalgae species. (EAA: Essential Amino Acid, NEAA: Non-Essential Amino Acid, Chaeto: *Chaetoseros* sp., Iso: *Isochrysis* sp., Sk: *Skeletonema* sp., Nanno: *Nanochloropsis* sp., Tetra: *Tetraselmis* sp.).

**Fig. 4:**
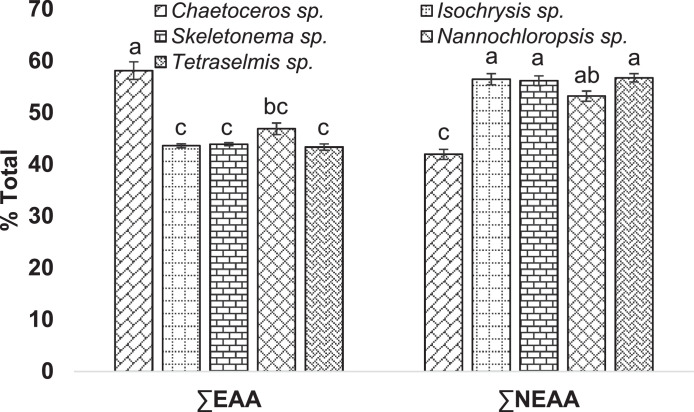
Amino acid content (% Total) in the cultured microalgae species. Different letters used in each category demonstrate the significant difference (*p* < 0.05). EAA: Essential amino acid, NEAA: Non-essential amino acid.

**Fig. 5:**
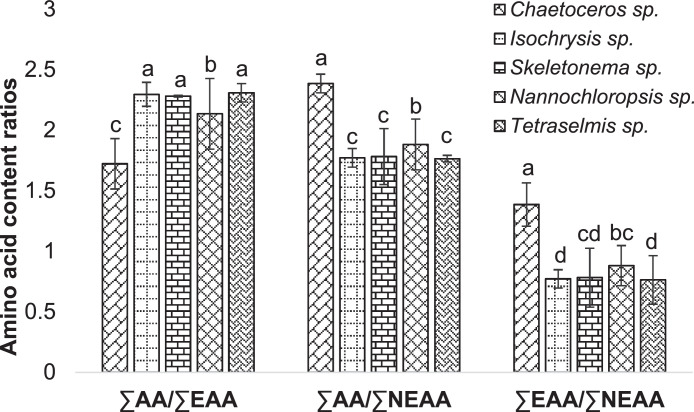
Amino acid content ratios in the cultured microalgae species. Different letters used in each category demonstrate the significant difference (*p* < 0.05). AA: Amino Acid, EAA: Essential Amino Acid, NEAA: Non-Essential Amino Acid.

This data shows the amino acid content of these selected microalgae species (Table 4, Figs. 3–5). The chromatogram for all the analyzed microalgae species are shown in Fig. 9.

**Table 4:** Amino acid content (% amino acid) in the cultured microalgae species.

			Species				
Parameters	Code name	Types	*Chaetoceros* sp.	*Isochrysis* sp.	*Skeletonema* sp.	*Nannochloropsis* sp.	*Tetraselmis* sp.
Histidine	HIS	EAA	3.40 ± 0.04	3.59 ± 0.12	2.46 ± 0.05	3.60 ± 0.06	4.19 ± 0.11
Isoleucine	ILE	EAA	6.97 ± 0.15	3.75 ± 0.02	4.87 ± 0.02	5.08 ± 0.15	3.30 ± 0.05
Leucine	LEU	EAA	10.35 ± 0.34	7.95 ± 0.05	9.50 ± 0.05	8.66 ± 0.35	7.81 ± 0.08
Lysine	LYS	EAA	3.64 ± 0.01	5.80 ± 0.08	4.46 ± 0.03	5.59 ± 0.03	5.74 ± 0.08
Methionine	MET	EAA	9.68 ± 0.06	2.28 ± 0.02	2.43 ± 0.06	4.55 ± 0.30	2.36 ± 0.05
Phenylalanine	PHE	EAA	5.80 ± 0.22	5.49 ± 0.01	6.50 ± 0.05	4.73 ± 0.09	5.60 ± 0.05
Threonine	THR	EAA	3.76 ± 0.28	5.69 ± 0.03	4.83 ± 0.01	4.68 ± 0.00	5.20 ± 0.00
Tyrosine	TYR	EAA	6.28 ± 0.26	2.51 ± 0.00	3.41 ± 0.04	3.26 ± 0.05	3.45 ± 0.12
Valine	VAL	EAA	8.20 ± 0.35	6.51 ± 0.04	5.41 ± 0.00	6.70 ± 0.07	5.66 ± 0.05
Alanine	ALA	NEAA	6.46 ± 0.17	7.78 ± 0.23	8.05 ± 0.04	8.87 ± 0.19	9.55 ± 0.08
Arginine	ARG	NEAA	3.94 ± 0.21	4.81 ± 0.08	5.71 ± 0.02	5.03 ± 0.08	5.30 ± 0.18
Aspartic acid	ASP	NEAA	9.00 ± 0.10	11.63 ± 0.06	12.12 ± 0.04	9.07 ± 0.08	10.74 ± 0.08
Glutamic acid	GLU	NEAA	8.87 ± 0.01	15.20 ± 0.26	12.92 ± 0.57	12.08 ± 0.23	14.14 ± 0.24
Glycine	GLY	NEAA	5.16 ± 0.29	6.13 ± 0.10	7.12 ± 0.10	6.62 ± 0.21	6.89 ± 0.17
Cysteine	CYS	NEAA	1.94 ± 0.08	1.60 ± 0.00	ND	1.16 ± 0.03	ND
Seronine	SER	NEAA	4.03 ± 0.09	5.92 ± 0.25	5.01 ± 0.07	4.21 ± 0.07	4.93 ± 0.01
Proline	PRO	NEAA	2.53 ± 0.04	3.35 ± 0.07	5.19 ± 0.11	6.11 ± 0.10	5.14 ± 0.05

Values are means ± standard error of duplicate measurements. EAA: Essential Amino Acid, NEAA: Non-Essential Amino Acid, ND: Not detected.

**Fig. 6:**
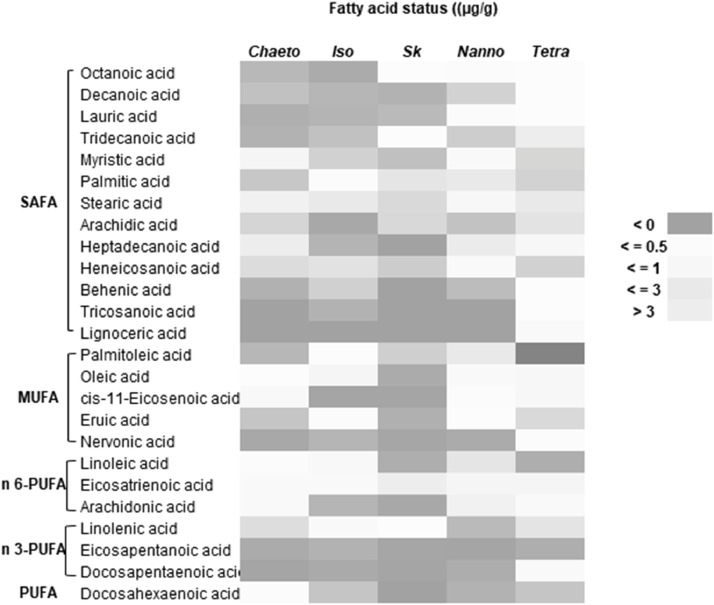
Heat-map presentation of fatty acid content in the cultured microalgae species. (SAFA: Saturated Fatty Acids, MUFA: Mono Unsaturated Fatty Acids, PUFA: Poly Unsaturated Fatty Acids, Chaeto: *Chaetoseros* sp., Iso: *Isochrysis* sp., Sk: *Skeletonema* sp., Nanno: *Nanochloropsis* sp., Tetra: *Tetraselmis* sp.).

**Fig. 7:**
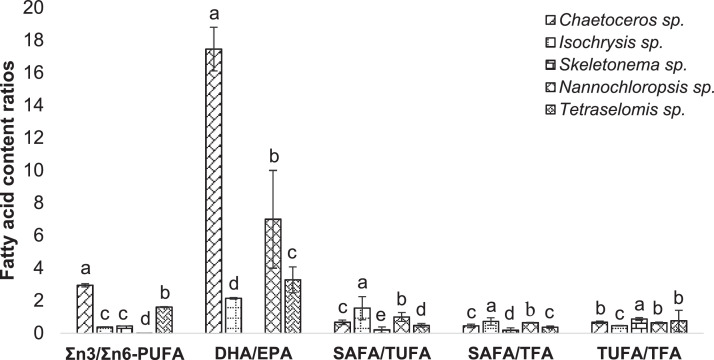
Fatty acid content ratios in the cultured microalgae species. Different letters used in each category demonstrate the significant difference (*p* < 0.05). SAFA: Saturated Fatty Acids, MUFA: Mono Unsaturated Fatty Acids, PUFA: Poly Unsaturated Fatty Acids, DHA: Docosahexaenoic Acid, EPA: Eicosapentaenoic Acid, TUFA: Total Unsaturated Fatty Acid, TFA: Total Fatty Acid.

**Fig. 8:**
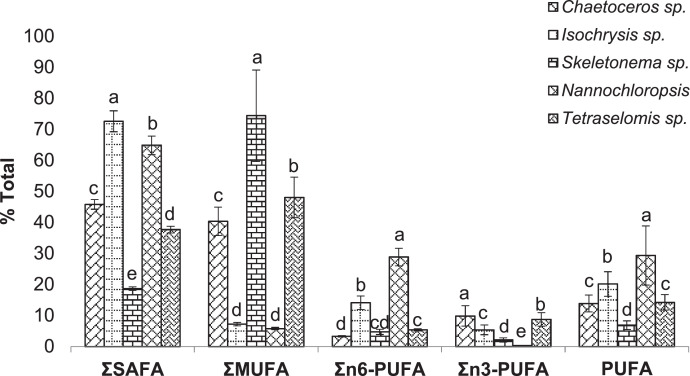
Fatty acid content (% total) in the cultured microalgae species. Different letters used in each category demonstrate the significant difference (*p* < 0.05). SAFA: Saturated Fatty Acids, MUFA: Mono Unsaturated Fatty Acids, PUFA: Poly Unsaturated Fatty Acids, DHA: Docosahexaenoic Acid, EPA: Eicosapentaenoic Acid, TUFA: Total Unsaturated Fatty Acid, TFA: Total Fatty Acid.

Finally, this data shows the fatty acid content of these selected microalgae species (Table 5, Figs. 6–8). The chromatogram for all the analyzed microalgae species are shown in Fig. 10.

**Table 5:** Fatty acid content (% fatty acid) in the cultured microalgae species.

			Species				
Carbon	Fatty acid	Types	*Chaetoceros* sp.	*Isochrysis* sp.	*Skeletonema* sp.	*Nannochloropsis* sp.	*Tetraselmis* sp.
C8:0	Octanoic acid	SAFA	0.09 ± 0.00	0.18 ± 0.00	2.12 ± 0.10	1.45 ± 0.12	0.47 ± 0.00
C10:0	Decanoic acid	SAFA	0.12 ± 0.00	0.36 ± 0.02	0.34 ± 0.04	0.42 ± 0.01	0.37 ± 0.31
C12:0	Lauric acid	SAFA	0.05 ± 0.00	0.31 ± 0.24	0.50 ± 0.06	0.77 ± 0.01	0.38 ± 0.01
C13:0	Tridecanoic acid	SAFA	0.06 ± 0.00	0.55 ± 0.04	1.89 ± 0.70	0.38 ± 0.08	0.28 ± 0.01
C14:0	Myristic acid	SAFA	2.51 ± 0.09	0.86 ± 0.08	7.68 ± 0.06	2.35 ± 0.34	0.19 ± 0.03
C16:0	Palmitic acid	SAFA	16.27 ± 0.11	3.61 ± 0.01	3.00 ± 0.06	13.26 ± 0.72	6.45 ± 0.22
C18:0	Stearic acid	SAFA	4.01 ± 0.42	27.13 ± 1.10	1.15 ± 0.27	4.17 ± 0.25	6.03 ± 0.70
C20:0	Arachidic acid	SAFA	12.15 ± 1.79	0.11 ± 0.00	1.12 ± 0.71	38.28 ± 4.39	7.14 ± 4.18
C17:0	Heptadecanoic acid	SAFA	0.30 ± 0.00	0.31 ± 0.16	ND	0.67 ± 0.44	1.85 ± 0.14
C21:0	Heneicosanoic acid	SAFA	10.18 ± 0.42	37.99 ± 1.10	0.89 ± 0.35	2.84 ± 1.27	12.43 ± 0.81
C22:0	Behenic acid	SAFA	0.06 ± 0.06	0.84 ± 0.63	ND	0.24 ± 0.24	0.49 ± 0.04
C23:0	Tricosanoic acid	SAFA	ND	0.31 ± 0.12	ND	ND	0.35 ± 0.02
C24:0	Lignoceric acid	SAFA	ND	ND	ND	ND	1.25 ± 0.34
C16:1	Palmitoleic acid	MUFA	21.26 ± 0.60	2.17 ± 0.07	73.80 ± 1.52	0.63 ± 0.26	34.64 ± 1.24
C18:1	Oleic acid	MUFA	0.37 ± 0.01	1.51 ± 0.10	0.20 ± 0.20	2.19 ± 0.52	0.32 ± 0.01
C20:1	cis-11-Eicosenoic acid	MUFA	1.60 ± 0.09	0.03 ± 0.03	0.04 ± 0.02	1.59 ± 1.11	1.95 ± 0.12
C22:1	Eruic acid	MUFA	17.09 ± 0.21	3.22 ± 0.13	0.28 ± 0.20	1.33 ± 0.83	10.42 ± 0.64
C24:1	Nervonic acid	MUFA	0.02 ± 0.01	0.34 ± 0.05	0.06 ± 0.01	0.09 ± 0.05	0.72 ± 0.22
C18:2n-6	Linoleic acid	n6-PUFA	0.62 ± 0.04	7.33 ± 0.19	3.06 ± 0.00	14.88 ± 10.38	1.39 ± 0.05
C20:3n-6	Eicosatrienoic acid	n6-PUFA	1.42 ± 0.18	6.44 ± 0.16	1.56 ± 0.44	5.32 ± 3.26	2.56 ± 0.18
C20:4n-6	Arachidonic acid	n6-PUFA	1.32 ± 0.35	0.35 ± 0.35	0.15 ± 0.07	8.66 ± 7.98	1.47 ± 0.08
C18-3n-3	Linolenic acid	n3-PUFA	9.83 ± 0.16	4.96 ± 0.20	2.05 ± 1.37	0.20 ± 0.01	7.22 ± 0.34
C20:5n-3	Eicosapentanoic acid	n3-PUFA	0.04 ± 0.02	0.30 ± 0.18	0.08 ± 0.05	0.02 ± 0.00	0.04 ± 0.02
C22:5n-3	Docosapentaenoic acid	n3-PUFA	0.01 ± 0.00	0.15 ± 0.01	0.04 ± 0.01	0.09 ± 0.07	1.47 ± 0.09
C22:6n-3	Docosahexaenoic acid	PUFA	0.65 ± 0.02	0.63 ± 0.03	ND	0.14 ± 0.01	0.13 ± 0.03

Values are mean ± standard error of duplicate measurements. SAFA: Saturated Fatty Acids, MUFA: Mono Unsaturated Fatty Acids, PUFA: Poly Unsaturated Fatty Acids.

**Fig. 9:**
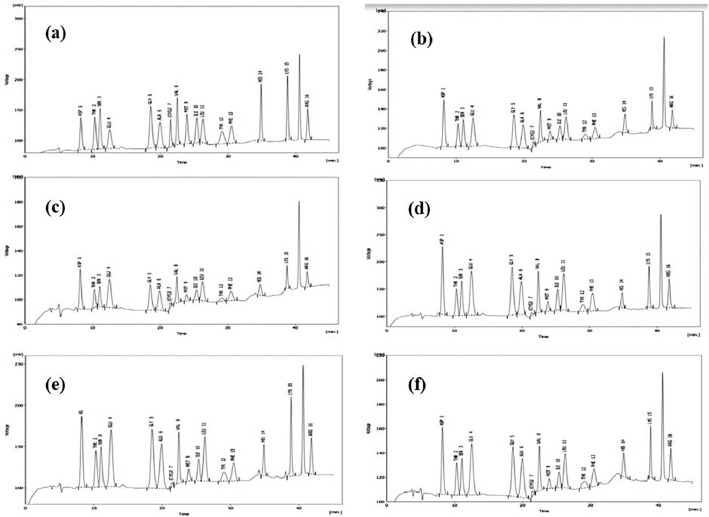
Chromatogram of amino acid analysis of all samples. Standard (H-G INJ1) (a) *Chaetoceros* sp. (b) *Isochrysis* sp. (c) *Skeltonema* sp. (d) *Nannochloropsis* sp. (e) *Tetraselmis* sp. (f).

**Fig. 10:**
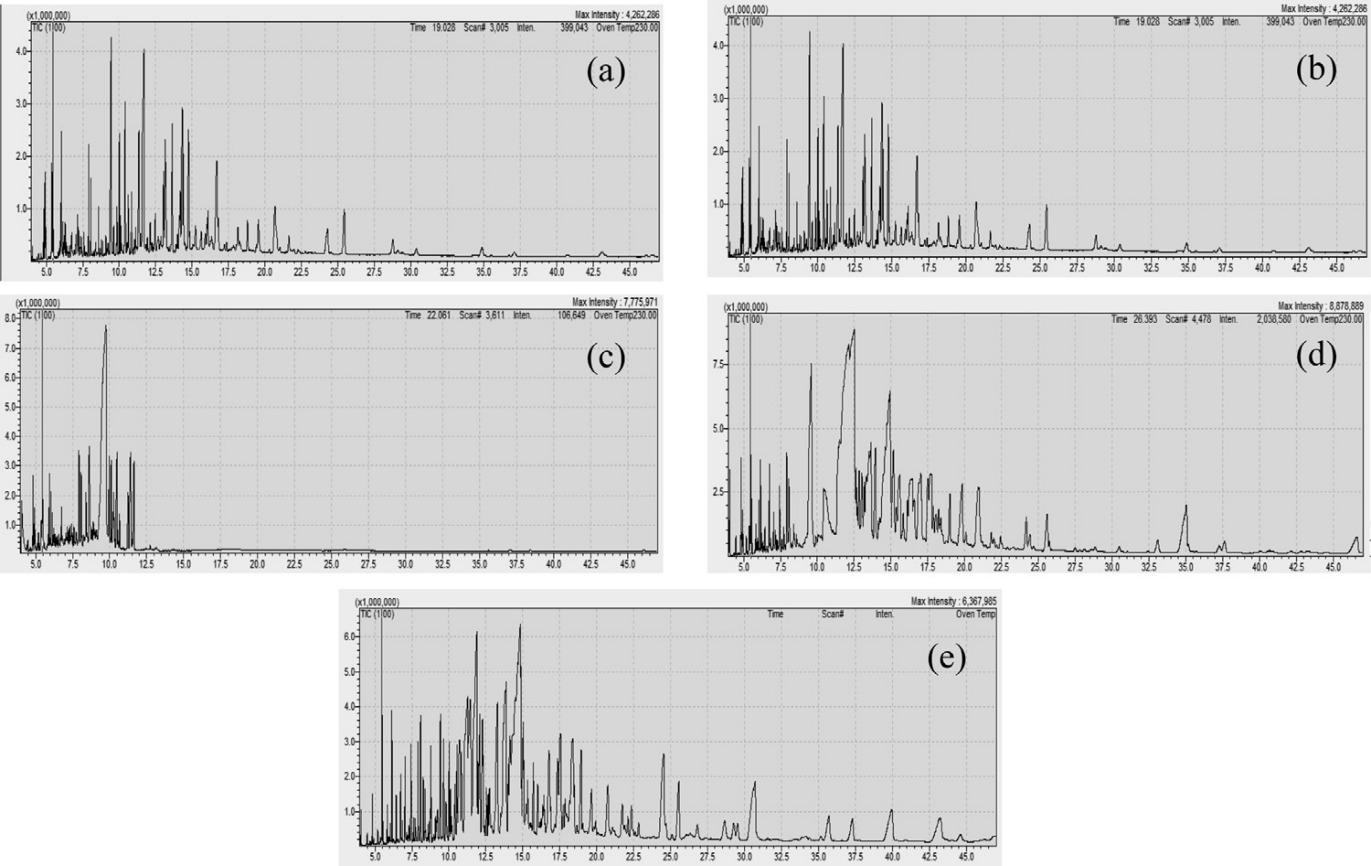
Chromatogram of fatty acid analysis of all samples. *Chaetoceros* sp. (a) *Isochrysis* sp. (b) *Skeltonema* sp. (c) *Nannochloropsis* sp. (d) *Tetraselmis* sp. (e).

## 2 Materials and Methods

### 2.1 Standard F/2 Guillard's Medium Preparation

Guillard's medium contains different micronutrients, trace elements and vitamins (Table 6). Initially seawater was collected from the Cox's Bazar coast of Bay of Bengal, Bangladesh to prepare pure medium for culturing the selected microalgae. Then the collected seawater was stabilized, filtered and autoclaved (15 lbs./inch^2^ for 15 min). To prepare 1 L medium 2 mL of solution A and B (in case of diatoms), 1 mL of solution C and D were added into the previously sterilized 28–30 ppt seawater (Table 6).

**Table 6:** Constituents of F/2 Guillard's culture medium stock solution.

Name of the Chemicals	Quantity
(A) Main mineral solution	
NaNO_3_	84.15 g
Na_2_MoO_4_.2H_2_O	6.0 g
FeCl_3_.6H_2_O	2.90 g
Na_2_EDTA.2H_2_O	10.0 g
Dissolving in deionized/distilled water and make the volume 1 L.	
(B) Silicate solution	
Na_2_SiO_3_.9H_2_O	33.0 g
Dissolving in deionized/distilled water and make the volume 1 L.	
(C) Trace metal solution	
CuSO_4_.5H_2_O	1.96 g
ZnSO_4_.7H_2_O	4.40 g
Ma_2_MnO_4_.2H_2_O	1.26 g
MnCl_2_.4H_2_O	36.0 g
CoCl_2_.6H_2_O	2.0 g
Dissolving in deionized/distilled water and make the volume 1 L.	
(D) Vitamin solution	
B_1_	0.4 g
B_12_	0.002 mg
Biotin	0.1 mg
Dissolving in deionized/distilled water and make the volume 1 L.	

### 2.2 Collection of Selected Microalgae

These five (5) commercially important marine microalgae (*Chaetoceros* sp.; *Isochrysis* sp.; *Skeletonema* sp.; *Nannochloropsis* sp.; and *Tetraselmis* sp.) were collected from previously isolated and stocked samples of the Live feed laboratory of Marine Fisheries and Technology Station, Bangladesh Fisheries Research Institute, Cox's Bazar, Bangladesh. These collected stocks were cultured and maintained at a standard temperature range (25 ± 2 ⁰C) at 150 µEm^−2^S^−1^ light intensity for 24 h with continuous sterile aeration [2,3].

### 2.3 Determination of the Morphological Traits

All the species were observed under a microscope using optimum magnification and fining. The morphological traits (color, shape, length, flagella, motility, and chain formation) were determined using a computerized light microscope Leica DM1000.

### 2.4 Determination of Growth Dynamics

All the collected species were cultured in F/2 Guillard's medium (triplicate replications), maintaining optimum standard parameters according to Islam et al. [3]. Cell density and biomass were measured to understand the growth pattern. Cell density was measured in everyday at a fix time. Haemocytometer (Hawksley AC1000, UK) was used to determine the cell density with following the method described by Lavens and Sorgeloos [4]. To determine dried biomass, initially 1 mL aliquot culture for each flask was filtered using a pre-weighted GF/C glass fibre filter paper and dried at 100 ⁰C for 4 h. Then, the filter paper was cooled for 15 min in a desiccator and reweighted. Finally, the dried biomass was calculated from the weight differences.

In addition, growth rates were determined according to the following equation,

$$K^{\prime }=\;ln\frac{N2}{N1}\;/\;T2-\;T1$$

where, *N*_2_ and *N*_1_ = Cell density at time T_1_ and T_2_, respectively.

### 2.5 Determination of the Physico-Chemical Parameters of the Cultured Medium

The physico-chemical parameters of the culture medium were determined at every alternate day from day 0 to stationary phase for every species separately. Temperature, light intensity, salinity, dissolve oxygen, and pH of the culture medium were measured using glass thermometer, lux meter, refractometer, DO meter and pH meter respectively. Total ammonium nitrogen (TAN), and soluble reactive phosphorus (SRP) were measured according to the method suggested by Parsons et al. [5]. In contrast, Nitrite nitrogen (NO_2_-N_2_) was determined according to the method suggested by Kitamura et al. [6]. Observe sure all of the equipment was calibrated before usage.

### 2.6 Mass Culture of Microalgae

For biomass preparation mass culture was done for all the species in a larger scale in a 20 L transparent food grade plastic jar using F/2 Guillard's medium. The cultures were scaled up gradually from a 10 mL test tube to 20 L plastic jar with following the protocol described by Amira et al. [7]. Each species was harvested at their stationary phase through centrifugation and oven dried at 60 ⁰C temperature for 12 h. Later the species were preserved at 4 ⁰C in normal refrigerated conditions for further analysis.

### 2.7 Amino Acid Determination

Amino acids were determined according to Moore and Stein [8] method with slight modification. Initially, 1 g dried biomass of each microalgae was hydrolyzed in 25 mL previously prepared acidic hydrolysis (6 M HCl + 0.1% phenol) solution at 110 ± 2 ⁰C temperature for 24 h. After cooling, the samples were stabilized using little amount of SDB/Na (Sample Dilution Buffer). Then the pH of the samples was adjusted in between 2.1 and 2.3 with using basic neutralization agent. Finally, the hydrolysates were transferred into the injection vials through filtration and diluted using SDB/Na.

The analysis was done using SYKAM S 433 amino acid analyzer equipped with UV detector. Nitrogen gas was used as carrier gas with maintaining 0. 5 mL/minute flow rate at 60 ⁰C temperature, where the reproducibility was 3%. AA-S-18 Sigma-Aldrich, Germany standard wease used for amino acids concentration determination. The amino acids contents were expressed as mg/g, which were finally converted in % of total amino acids.

### 2.8 Fatty Acid Determination

Fatty acids were determined according to Prato et al. [9]. Initially, lipid was extracted from all the dried biomasses using Soxhlet apparatus. The sample were placed in a thimble paper for running the cycle. Acetone was used as solvent. Standard temperature 60 ⁰C was maintained during lipid extraction. The extracted lipid was collected after complete extraction. Then fatty acids of methyl esters (FAMEs) were prepared for gas chromatography. The gas chromatography mass spectrophotometry analysis was run using a GCMS-QP2020 (Shimadzu, Japan), equipped with flame ionization detector. The 30 m long capillary column with 0.25 mm diameter and 0.15 µm thickness was used for FAMEs separation. Helium gas was used as carrier gas with maintaining 1.42 mL/minute flow rate at 180–280 ⁰C temperature at 5 ⁰C/ minute. FAME mix C8-C24; Sigma-Aldrich, Germany standard were used for FAMEs identification with comparing the retention time. The fatty acids content were expressed as µg/g which were finally converted in % of total fatty acids.

### 2.9 Statistical Analysis

Mean, standard error of mean (SE = σ /√n) of the data were calculated using MS excel (v. 2016). One-way multivariate analysis was performed to determine whether there is any significance difference among the species. The post hoc test was performed at 5% significance using IBM SPSS (v. 26.0).

## Ethics Statement

These data were collected complying ARRIVE guidelines. Authors skipped obtaining legal authority's ethical consent before beginning the data collection process because microalgae are not protected by any regulations or laws in Bangladesh.

## CRediT authorship contribution statement

**Jakia Hasan:** Conceptualization, Investigation, Supervision, Methodology, Writing – review & editing. **Zahidul Islam:** Conceptualization, Investigation, Methodology, Data curation, Writing – original draft, Writing – review & editing. **Ahmad Fazley Rabby:** Methodology, Writing – review & editing. **Saima Sultana Sonia:** Methodology, Writing – review & editing. **Md. Aktaruzzaman:** Methodology, Writing – review & editing. **Turabur Rahman:** Methodology, Writing – review & editing. **Shafiqur Rahman:** Supervision, Funding acquisition, Writing – review & editing. **Yahia Mahmud:** Supervision, Funding acquisition, Writing – review & editing.

## Declaration of Competing Interest

None.

## Data Availability

Dataset describing the growth pattern, amino acid and fatty acid profile of five indigenous marine microalgae species of Bangladesh (Original data) (Mendeley Data).
